# Human nucleoli comprise multiple constrained territories, tethered to individual chromosomes

**DOI:** 10.1101/gad.348234.121

**Published:** 2021-04-01

**Authors:** Hazel Mangan, Brian McStay

**Affiliations:** Centre for Chromosome Biology, School of Natural Sciences, National University of Ireland Galway, Galway H91 W2TY, Ireland

**Keywords:** nucleolus, human acrocentric chromosome, nucleolar organizer region (NOR), ribosomal DNA (rDNA), ribosome biogenesis

## Abstract

It is unknown how ribosomal gene (rDNA) arrays from multiple chromosomal nucleolar organizers (NORs) partition within human nucleoli. Exploration of this paradigm for chromosomal organization is complicated by the shared DNA sequence composition of five NOR-bearing acrocentric chromosome p-arms. Here, we devise a methodology for genetic manipulation of individual NORs. Efficient “scarless” genome editing of rDNA repeats is achieved on “poised” human NORs held within monochromosomal cell hybrids. Subsequent transfer to human cells introduces “active” NORs yielding readily discernible functional customized ribosomes. We reveal that ribosome biogenesis occurs entirely within constrained territories, tethered to individual NORs inside a larger nucleolus.

## Abstract (precis)

In this study, Mangan et al. investigated how ribosomal gene (rDNA) arrays from multiple chromosomal nucleolar organizers (NORs) partition within human nucleoli, which is complicated by the shared DNA sequence composition of five NOR-bearing acrocentric chromosome p-arms. They present a methodology for genetic manipulation of individual NORs and reveal that ribosome biogenesis occurs entirely within constrained territories, tethered to individual NORs inside a larger nucleolus.

Nucleoli, sites of ribosome biogenesis, form around NORs located on the p-arms of five human acrocentric chromosomes, HSA13, HSA14, HSA15, HSA21, and HSA22 (Fig. 1A; McStay 2016). Sequences of rDNA arrays together with proximal and distal junctions (PJs and DJs) are shared between all five acrocentrics (Floutsakou et al. 2013; van Sluis et al. 2019). DJs are functional NOR elements, embedded in perinucleolar heterochromatin (PNH) (Floutsakou et al. 2013). During metaphase, NORs are bookmarked by UBF (upstream binding factor), a nucleolar HMG-box protein that binds extensively across rDNA arrays (Grob et al. 2014). As cells exit anaphase, transcription by RNA polymerase I (RNA Pol I) resumes and nucleoli form around individual NORs (Hernandez-Verdun 2011; van Sluis et al. 2020). These coalesce into mature nucleoli comprising three distinct compartments, reflecting the stages of ribosome biogenesis (Raška et al. 2006). Fibrillar center (FC) units contain one or a few UBF-loaded rDNA repeats (Yao et al. 2019). Transcription occurs at the interface between FCs and the surrounding dense fibrillar component (DFC) formed on nascent transcripts. FC/DFC units are embedded in the granular component (GC) where released pre-rRNAs are processed and assembled into ribosomal subunits.

**Figure 1.**
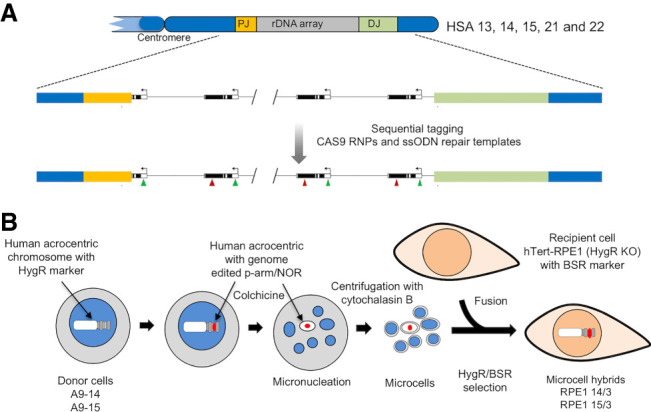
Sequence tagging of NORs and transfer to human cells. (*A*) Organization of NORs on acrocentric p-arms, depicting rDNA arrays and proximal and distal junctions (PJs and DJs). A representation of a sequenced tagged NOR is shown *below*. (*B*) MMCT workflow for transfer of human chromosomes with edited NORs from A9 hybrid donor cells into recipient hTERT-RPE1 Hyg^−ve^ cells.

Nucleoli are a paradigm for specific associations between multiple chromosome territories to form nuclear bodies (Misteli 2020). They are major spatial genome organizers, providing dominant positional cues for up to 10 chromosomes (van Sluis et al. 2020). Potential advantages of gathering multiple NORs in a single nucleolus include enhanced ribosome biogenesis efficiency and isolation from incompatible nuclear activities. However, blending the most transcriptionally active regions of the genome from multiple chromosomes within a single nuclear body represents a major threat to their genome stability. Here, we address this issue by examining how individual NORs, together with their pre-rRNA products, organize within nucleoli.

## Results and Discussion

Human acrocentric chromosomes held within mouse A9 monochromosomal somatic cell hybrids bear transcriptionally silent NORs due to cross-species incompatibility of transcription factor SL1 (Sullivan et al. 2001). Nevertheless, these NORs are held in a “poised” state due to extensive UBF binding and can be reactivated by ectopic human SL1 (van Sluis et al. 2019). We reasoned that sequence tagging of human rDNA in hybrids by CRISPR/Cas9 genome editing, combined with chromosome transfer into human cells, would enable visualization of single “active” NORs within a human nucleolus (Fig. 1).

Microcell-mediated chromosome transfer (MMCT) is a proven method for shuttling chromosomes from donor to recipient cells (Meguro-Horike and Horike 2015). Under prolonged exposure to colchicine, A9 cells undergo micronucleation. Centrifugation in the presence of cytochalasin B leads to the production of microcells that are then fused to recipient cells (Fig. 1B). Key to successful MMCT, donor chromosomes and recipient cells contain separate selectable markers. A hygromycin resistance (HygR) cassette on human acrocentric chromosomes in A9 hybrids provides donor chromosome selection. The human recipient cell line of choice was human telomerase-immortalized retinal pigmented epithelial cells (hTERT-RPE1) that are karyotypically normal, have intact DNA damage checkpoints, and well-characterized NORs (van Sluis et al. 2020). Prior to using hTERT-RPE1, we disrupted the HygR ORF and introduced a blasticidin resistance (Bsr) cassette to facilitate MMCT double selection (Supplemental Fig. S1). We initially transferred HSA15 present in hybrid A9-15 into hTERT-RPE1 Hyg^−ve^ cells. Validation of the resulting cell line revealed that, other than the presence of an extra HSA15, the karyotype remains unaltered (Supplemental Fig. S2). Moreover, growth characteristics of these cells are indistinguishable from the hTERT-RPE1 Hyg^−ve^ recipient line.

**Figure 2.**
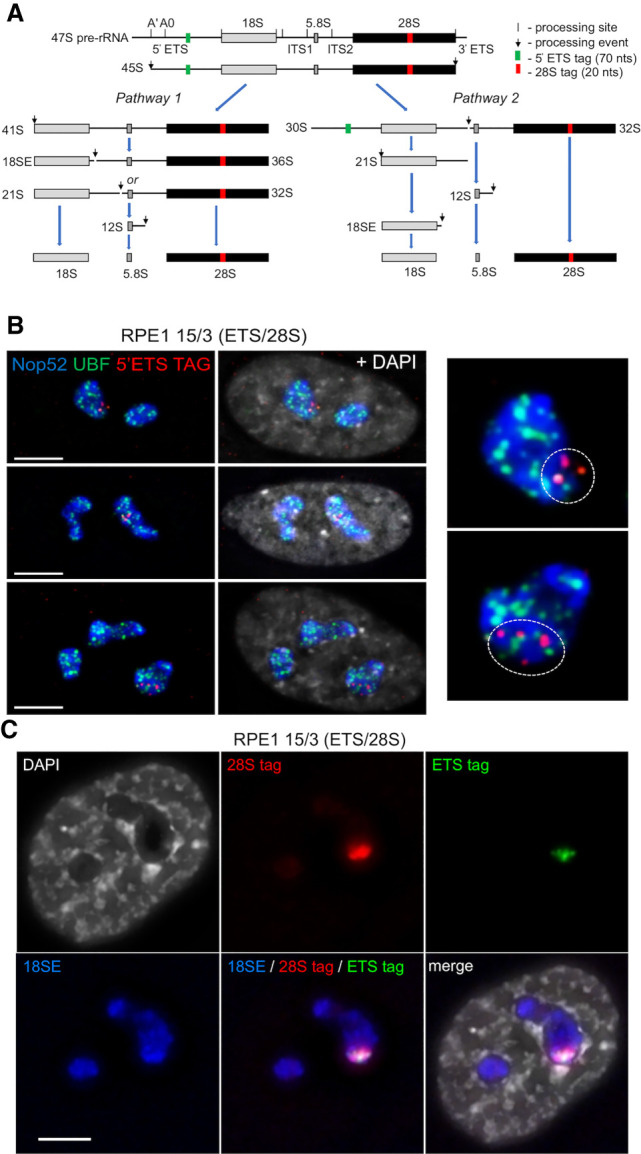
Genome-edited NORs and pre-rRNAs occupy constrained territories within nucleoli. (*A*) Alternate pre-rRNA processing pathways, showing positions of 5′ETS and 28S sequence tags. (*B*) SABER-FISH on 15/3(ETS/28S) cells using 5′ETS tag probes, combined with UBF and Nop52 antibody staining. (*Left*) Tagged NOR-territories are indicated by a white dotted line on enlarged images of single nucleoli. (*C*) RNA-FISH with probes recognizing 5′ETS and 28S tags and 18SE pre-rRNA reveals that nascent and released pre-rRNAs from the tagged NOR in 15/3(ETS/28S) remain within a defined NOR territory. Scale bars, 5 µm.

Sequential tagging of 5′ETS and 28SrRNA on the HSA15 rDNA array in the hybrid line A9-15 was carried out using CRISPR/Cas9 RNPs with single-strand oligodeoxynucleotides (ssODNs) as the homology-directed repair (HDR) donor templates (Supplemental Fig. S3; Supplemental Table S1). SsODNs are the preferred choice for introducing short sequence tags as they avoid undesired integration events normally associated with double-strand DNA (dsDNA) donor templates (Yang et al. 2013). A 70-nt ETS tag provides a mark for nascent transcripts in the DFC, while a 20-nt 28S tag should be present in both the DFC and in released pre-rRNAs within the GC (Fig. 2A). Appearance of cytoplasmic tagged 28SrRNAs supports functionality in translation. Importantly, sequences at tag locations in human pre-rRNA differ sufficiently from mouse, such that guide RNAs (gRNAs) will not recognize host cell rDNA arrays (Supplemental Fig. S4). In cell line A9-15(ETS/28S), ∼50% of repeats are ETS-tagged and >90% of repeats 28S-tagged. Sequencing reveals that all tagged repeats have undergone “scarless” genome editing while most nontagged repeats have indels of 1 or 2 nt, indicative of double-strand break (DSB) repair by nonhomologous end-joining (NHEJ) (Supplemental Fig. S4C).

Following MMCT of double-tagged HSA15, generating 15/3(ETS/28S) (Supplemental Fig. S5A), we assessed the nucleolar distribution of tagged rDNA by SABER (signal amplification by exchange reaction) FISH (Kishi et al. 2019). A SABER probe visualizing 5′ETS DNA tags in the edited HSA15 NOR, combined with UBF and Nop52 antibodies to visualize nucleolar FCs and GCs, revealed that tagged repeats occupy a distinct territory within larger nucleoli (Fig. 2B).

Next, we determined the distribution of tagged pre-rRNA processing intermediates deriving from the 15/3(ETS/28S) genome-edited NOR. RNA-FISH was performed using fluorescent oligonucleotide probes recognizing each tag, combined with a nucleolar marker probe recognizing the 3′ end of 18SE pre-rRNA (Fig. 2C). Punctate 5′ETS tag hybridization signals are observed within a larger subnucleolar region identified by the 28S tag probe. As the majority of the 28S tag signal derived from released pre-rRNA species, we conclude that the engineered NOR generates its own distinct GC compartment. Quantitation of data from this experiment reveals that tagged NORs occupy, on average, 23.5% ± 15.4% (1 standard deviation, SD) of the area in the nucleoli in which they reside and 13.8% ± 7.0% (1 SD) of the combined nucleolar area in each cell (Supplemental Fig. S6). As the edited HSA15 NOR is one of 11 acrocentric chromosomes in these cells, these results are in line with expectations. Thus, it appears that individual rDNA arrays and their derived pre-rRNA processing intermediates are constrained within NOR territories.

**Figure 3.**
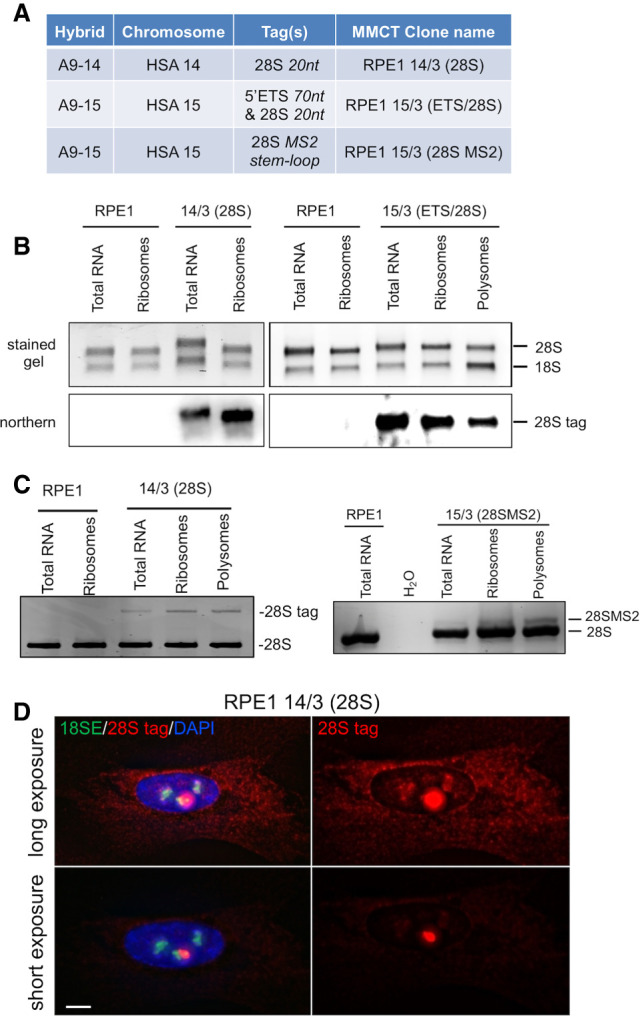
Genome-edited rDNA arrays yield functional ribosome subunits. (*A*) Hybrids used in genome editing, sequence tags, and post-MMCT cell lines. (*B*) Northerns of total RNA and RNA isolated from ribosome and polysome fractions of RPE1, 14/3(28S), and 15/3(ETS/28S) cells, using a 28S-tag DIG-labeled probe. (*C*) RT-PCR reveals the proportion of 28SrRNA deriving from edited NORs in 14/3(28S) and 15/3(28SMS2) ribosome and polysome fractions. (*D*) RNA-FISH identifies cytoplasmic tagged 28SrRNA in 14/3(28S) cells. Nucleoli are visualized with an 18SE oligo probe. Scale bar, 5 µm.

To strengthen our findings, we generated A9-14(28S) and A9-15(28SMS2) by tagging >90% of 28SrRNA coding sequences in the hybrids A9-14 and A9-15. The 28SMS2 tag encodes a 19-nt stem-loop binding site for the bacteriophage MS2 coat protein (MCP) (Supplemental Fig. S4B; Weil et al. 2010). Subsequent MMCT generated RPE1 14/3(28S) and 15/3(28SMS2) (Fig. 3A; Supplemental Fig. S5).

We reasoned that insertion of short sequence tags in expansion segment ES10L of 28SrRNA coding would disrupt neither biogenesis nor function of large ribosome subunits (Anger et al. 2013). Northern blots of total cellular RNA, and RNA extracted from purified cytoplasmic ribosomes prepared from 14/3(28S), 15/3(ETS/28S), and control RPE1 cells, were probed using a digoxygenin (Dig)-labeled oligonucleotide recognizing tagged 28SrRNA. They revealed that edited rDNA arrays yield mature tagged 28SrRNAs appearing in cytoplasmic ribosomes. Functionality is confirmed by their presence in polysomes (Fig. 3B). Reverse transcriptase-PCR (RT-PCR) showed that 5%–10% of ribosomes are assembled around tagged 28SrRNA in 14/3(28S) and 15/3(28SMS2) cells (Fig. 3C). As tagged NORs represents one out of 11 NORs in these cell lines, these levels indicate full functionality of genome-edited NORs. Further validation came from RNA-FISH, using a 28S tag and the 18SE probes in 14/3(28S) cells (Fig. 3D). In longer exposures, accumulation of cytoplasmic tagged 28SrRNA is clearly observed. Lower exposures reveal the subnucleolar territory associated with expression from the tagged NOR. RNA-FISH performed on cocultured 14/3(28S) and recipient RPE1 cells provided a control for probe specificity (Supplemental Fig. S7). Combined, these data establish that genome-edited NORs produce functional ribosome subunits entirely within a constrained NOR territory.

Following inhibition of RNA Pol I transcription by actinomycin D (AMD) or in response to rDNA DSBs, nucleoli undergo a rapid and profound reorganization (van Sluis and McStay 2015; Mangan et al. 2017). FC and DFC components coalesce and move together with rDNA to the nucleolar periphery, forming caps on the surface of a GC nucleolar interior. Mature nucleoli have multiple caps, each deriving from a single NOR and forming adjacent to its linked DJ sequence embedded in the PNH (Floutsakou et al. 2013).

**Figure 4.**
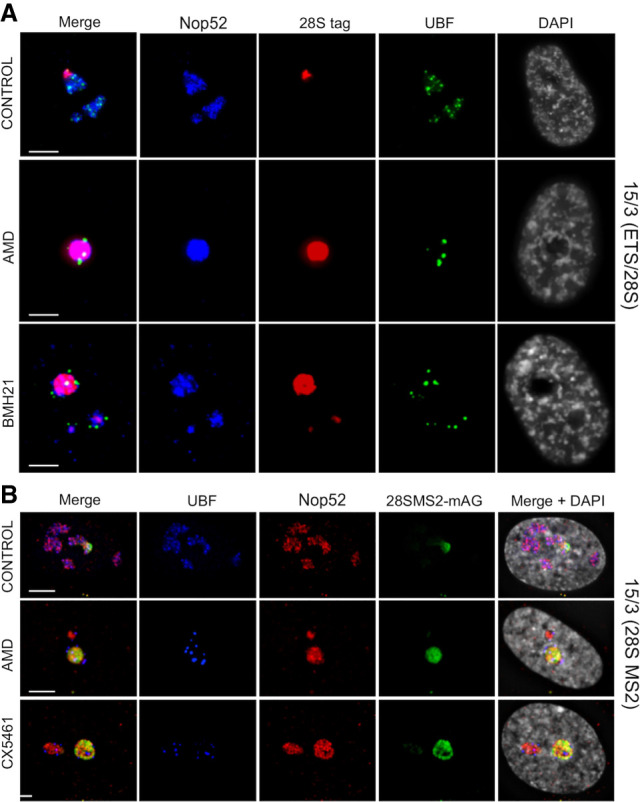
Blending of territories during nucleolar stress. (*A*) Immuno-RNA-FISH was performed on 15/3(ETS/28S) cells either untreated or treated with inhibitors of RNA Pol I transcription, AMD and BMH21. 28S tag was visualized using an oligonucleotide probe (red). Nucleoli were visualized using antibodies against UBF (green) and Nop52 (far red, pseudocolored blue). DAPI is pseudocolored gray. (*B*) 15/3(28S MS2) cells expressing mAG-MS2 either untreated or treated with AMD and CX5461. Nucleoli were visualized using antibodies against UBF (far red, pseudocolored blue) and Nop52 (red). DAPI is pseudocolored gray. Live-cell imaging data of these cells treated with AMD is presented in Supplemental Figure S9. Scale bars, 5 µm.

Against this backdrop, we asked whether NOR territories are maintained under conditions of nucleolar stress. 15/3(ETS/28S) cells were treated with AMD and another inhibitor of RNA Pol I transcription, BMH21 (Peltonen et al. 2014). In RNA immuno-FISH, a probe against tagged 28SrRNA was combined with Nop52 and UBF antibody staining to reveal nucleolar GC and FC, respectively (Fig. 4A). Results from untreated cells confirm that tagged NORs occupy distinct nucleolar territories. In RNA Pol I inhibited cells, we observe that released pre-rRNA processing intermediates derived from tagged NORs have spread throughout the GC interior of mature nucleoli. Importantly, we can be certain that these nucleoli are comprised of multiple NORs, as judged by the presence of multiple UBF positive caps. Similar results were obtained using 14/3(28S) cells treated with AMD, BMH21, and CX5461, another inhibitor of RNA Pol I (Supplemental Fig. S8; Bywater et al. 2012). NOR territories can also be observed in 15/3(28SMS2) cells expressing a monomeric Azami Green-MCP fusion protein (mAG-MS2) (Fig. 4B). During nucleolar stress, mAG-MS2 spreads throughout the nucleolar interior. Live-cell imaging reveals that blending of territories initiates 10 min after AMD addition and is complete by 45 min (Supplemental Fig. S9). These data combined illustrate that, on withdrawal of rDNA and associated FC and DFC components to caps during nucleolar stress, constraints that maintain NOR territories are removed and GCs derived from individual NORs blend freely.

**Figure 5.**
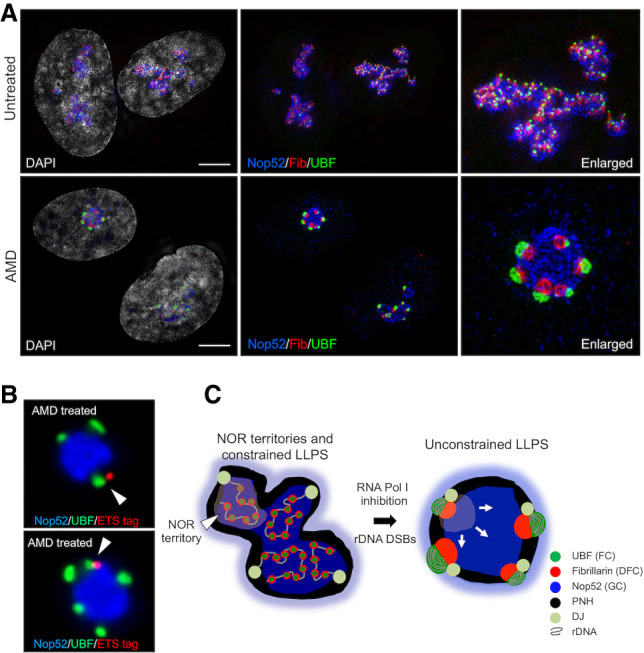
Nucleolar reorganization under stress. (*A*) Untreated (*top*) and AMD-treated (*bottom*) hTERT-RPE1 cells stained with antibodies against UBF, fibrillarin, and Nop52 and analyzed by SIM. Enlarged images of single nucleoli are shown at the *right*. (*B*) SABER-FISH performed on AMD-treated 15/3(ETS/28S) cells using 5′ETS tag probes, combined with UBF and Nop52 antibody staining. Nucleolar caps deriving from the genome-edited HSA15 are indicated by arrowheads. (*C*) A model for nucleolar organization in normal growth conditions and under nucleolar stresses such as direct inhibition of RNA Pol I transcription or introduction of rDNA DSBs. (DJ) Distal junction, (PNH) perinucleolar heterochromatin. Scale bars, 5 µm.

To further investigate the organization of nucleoli under stress, we used structured illumination microscopy (SIM) to compare the distribution of UBF, fibrillarin, and Nop52, marker proteins for FC, DFC, and GC respectively, before and after AMD treatment (Fig. 5A). The nucleolar morphology of untreated cells is clearly irregular, with many FC/DFC units embedded in the GC. Strikingly, SIM reveals that, in AMD treated cells, nucleolar caps are bipartite with clearly delineated FCs and DFCs. Notably, cap DFCs project into the GC interior while FCs project outward into the nucleoplasm. Indeed, it appears that stressed nucleoli adopt an inverted organization relative to their nonstressed state. The other striking morphological change is that the overall shape of stressed nucleoli has become more spherical, also visible above (Fig. 4; Supplemental Figs. S7, S8). SABER-FISH on AMD-treated 15/3(ETS/28S) cells reveals that tagged repeats from the genome-edited HSA15 are associated with a single nucleolar cap (Fig. 5B).

The current prevailing model proposes that the internal organization of nucleoli forms through liquid-liquid phase separation (LLPS) (Lafontaine et al. 2020). This model has been informed by the study of representative proteins from each of the nucleolar components and has taken little account of NORs, the chromosomal domains that direct nucleolar formation. Our findings indicate that NORs dominate nucleolar organization by establishing NOR territories, also suggested by the irregular shape of mature nucleoli. We establish that a “production line” of ribosome biogenesis intermediates, and the rDNA array from which they emerge, are constrained within a NOR territory. Although pre-rRNA processing intermediates are restricted to their NOR territory of origin, photodynamic experiments have clearly established that individual nucleolar polypeptides exchange freely between the nucleoplasm and nucleoli and likely between NOR territories (Dundr et al. 2002). Organization within individual NOR territories probably involves constrained LLPS. FC/DFC units are constrained by tethering to a single cluster or small clusters of rDNA repeats (Yao et al. 2019). We propose that some activity constrains the GC within a NOR territory. Proteins normally present in nucleoli that relocalize to the nucleoplasm under nucleolar stress are prime candidates. The proliferation marker Ki-67 is one such candidate (Kill 1996; Booth et al. 2014; Sun and Kaufman 2018).

In summary we propose that under normal growth conditions, NORs provide major organizational influences on shaping nucleoli, but that under nucleolar stress conditions, LLPS becomes the major organizational principle (Fig. 5C). We further propose that NOR territories shield rDNA arrays from inter-chromosomal entanglements during cell division and stress responses induced by rDNA DSBs (Hernandez-Verdun 2011; Potapova et al. 2019; van Sluis and McStay 2019). Finally, the ability to produce customized ribosomes establishes our methodology as a platform for future investigation of human ribosome biogenesis and function in human cells.

## Materials and methods

### Cell culture

A9 hybrids and hTERT-RPE1 cells were maintained as previously described (van Sluis et al. 2019). To inhibit RNA Pol I transcription, cells were treated for 1 h with 0.1 µg/mL actinomycin D (AMD) (Sigma), 1 µM BMH21 (Sigma), or 1 µM CX-5461 (Selleck).

### Tagging of rDNA arrays

For genome editing of rDNA arrays, 400 ng of gRNA (10 pmoles) were precomplexed with 1 µg (6 pmol) of Alt-R S.p. Cas9 nuclease V3 (Integrated DNA Technologies) for 10 min at room temperature. RNP complexes were electroporated into target cells using the neon transfection system (Invitrogen) in 10 µL tips using 100,000 cells, 500 ng puromycin mRNA, and 10 µM ssODN using the parameters “1400V 10 ms x 3.” Transfected cells were selected 2 h posttransfection using 10 µg/mL puromycin (Sigma) for 48 h. DNA was harvested from the cell pool for PCR analysis, and cells were seeded for single-cell clones. After 10–14 d, single-cell colonies were picked into 96-well plates. Five days to 7d later, colonies were screened using DirectLyse PCR (Ramlee et al. 2015). Positive clones were expanded.

### Microcell-mediated chromosome transfer (MMCT)

MMCT was performed essentially as previously described (Meguro-Horike and Horike 2015). For each experiment, six T-25 flasks of A9-14 or A9-15 cells at 80%–90% confluency were treated with 0.075 µg/mL demecolcine (Sigma) for 48 h. On the day of MMCT, the flasks were filled to the neck with prewarmed serum-free F-12 media supplemented with 10 µg/mL cytochalasin B (Sigma). Flasks were submerged in water and centrifuged in 500-mL bottles (Nalgene) at 10,000*g* for 1 h at 34°C. The microcell pellet was resuspended in serum-free F-12 media and filtered through nitrocellulose membrane filters (8 µm and 5 µm, respectively; Whatman) in 25-mm Swinnex filter holders (Millipore). Microcells were centrifuged at 1500*g* for 10 min. The pellet was resuspended in 2 mL of serum-free F-12 media containing 200 µg/mL phytohemagglutinin P (Sigma) and applied to a 70% confluent 60-mm dish of hTERT-RPE1 Hyg^−ve^ cells. Following 30-min agglutination at 37°C, media was removed and membrane fusion was facilitated by addition of 1 mL of PEG 1500 for 2 min. Cells were then extensively washed with serum-free F-12 media. Following 24-h incubation in complete media, MMCT fused cells were selected using 400 µg/mL hygromycin and 20 µg/mL blasticidin. Clones were visible 2–3 wk later.

### Fluorescent in situ hybridization (FISH)

Human rDNA intergenic spacer (IGS), DJ, and HSA15 α-satellite DNA FISH probes have been described previously (van Sluis et al. 2019). Mouse Cot-1 DNA was from Invitrogen. Probes were directly labeled by nick translation using Green 496 dUTP, Red 580 dUTP, or Far Red 650 dUTP (Enzo). Chromosome paints were prepared by inter-alu PCR (Liu et al. 1993) using genomic DNA prepared from monochromosomal hybrids as template with KAPA HiFi DNA polymerase (Kapa Biosystems) and primers AluPCR F/R (Supplemental Table S1). Chromosome paints were biotin-labeled using a BioPrime kit (Invitrogen) and visualized using Cy3 strepavidin (Rockland). All RNA-FISH probes were fluorescently labeled oligonucleotides (Supplemental Table S1).

DNA-FISH on metaphase spreads, 3D immuno-FISH, and RNA-FISH on interphase cells were performed as described previously (van Sluis et al. 2019, 2020). For RNA-FISH, 250 ng of oligonucleotide probe was used per slide. For immunoRNA-FISH, hybridization time was reduced to 1 h. After posthybridization washes, primary antibody was diluted in 10 mg/mL acetylated BSA and incubated with cells for 1 h at 37°C followed by three 15-min PBS washes. Secondary antibody was diluted in 10 mg/mL acetylated BSA and incubated for 40 min at 37°C followed by a further three 15-min PBS washes. Cells were then mounted in VectaShield (Vector Laboratories) and imaged immediately. Antibodies are listed in Supplemental Table S2.

Signal amplification by exchange reaction (SABER) FISH probes were prepared and used as described previously (Kishi et al. 2019). A primer exchange reaction (PER) was performed using a mixture of two primers, G2R26.1 and G2R26.2 (Supplemental Table S1), that recognize the 70-nt ETS tag. The catalytic hairpin h.26.26.ip and Clean.G oligonucleotides used in PER and the ATTO 565-labeled oligonucleotide 26*.565 used for visualization have been described previously (Kishi et al. 2019). Antibody staining was performed subsequent to SABER FISH.

### Imaging

Images from fixed cells and metaphase spreads were captured and processed as described previously (van Sluis et al. 2019). For live-cell imaging, cells were grown in complete F-12 media without phenol red in µ-Dish 35-mm microscopy dishes (Ibidi, 81156). Cells were stained with 100 nM SiR-DNA (Spirochrome AG) 1 h prior to the start of live-cell imaging. SIM images were captured using a DeltaVision OMX SR imaging system (GE Healthcare) equipped with an sCMOS camera (PCO-Tech). Image reconstruction was performed with softWoRx.

### Competing interest statement

The authors declare no competing interests.

## Supplementary Material

Supplemental Material

## References

[GAD348234MANC1] Anger AM, Armache JP, Berninghausen O, Habeck M, Subklewe M, Wilson DN, Beckmann R. 2013. Structures of the human and *Drosophila* 80S ribosome. Nature 497: 80–85. 10.1038/nature12104 23636399

[GAD348234MANC2] Booth DG, Takagi M, Sanchez-Pulido L, Petfalski E, Vargiu G, Samejima K, Imamoto N, Ponting CP, Tollervey D, Earnshaw WC, 2014. Ki-67 is a PP1-interacting protein that organises the mitotic chromosome periphery. Elife 3: e01641. 10.7554/eLife.01641 24867636 PMC4032110

[GAD348234MANC3] Bywater MJ, Poortinga G, Sanij E, Hein N, Peck A, Cullinane C, Wall M, Cluse L, Drygin D, Anderes K, 2012. Inhibition of RNA polymerase I as a therapeutic strategy to promote cancer-specific activation of p53. Cancer Cell 22: 51–65. 10.1016/j.ccr.2012.05.019 22789538 PMC3749732

[GAD348234MANC4] Dundr M, Hoffmann-Rohrer U, Hu Q, Grummt I, Rothblum LI, Phair RD, Misteli T. 2002. A kinetic framework for a mammalian RNA polymerase in vivo. Science 298: 1623–1626. 10.1126/science.1076164 12446911

[GAD348234MANC5] Floutsakou I, Agrawal S, Nguyen TT, Seoighe C, Ganley AR, McStay B. 2013. The shared genomic architecture of human nucleolar organizer regions. Genome Res 23: 2003–2012. 10.1101/gr.157941.113 23990606 PMC3847771

[GAD348234MANC6] Grob A, Colleran C, McStay B. 2014. Construction of synthetic nucleoli in human cells reveals how a major functional nuclear domain is formed and propagated through cell division. Genes Dev 28: 220–230. 10.1101/gad.234591.113 24449107 PMC3923965

[GAD348234MANC7] Hernandez-Verdun D. 2011. Assembly and disassembly of the nucleolus during the cell cycle. Nucleus 2: 189–194. 10.4161/nucl.2.3.16246 21818412 PMC3149879

[GAD348234MANC8] Kill IR. 1996. Localisation of the Ki-67 antigen within the nucleolus. Evidence for a fibrillarin-deficient region of the dense fibrillar component. J Cell Sci 109(Pt 6**)**: 1253–1263. 8799815 10.1242/jcs.109.6.1253

[GAD348234MANC9] Kishi JY, Lapan SW, Beliveau BJ, West ER, Zhu A, Sasaki HM, Saka SK, Wang Y, Cepko CL, Yin P. 2019. SABER amplifies FISH: enhanced multiplexed imaging of RNA and DNA in cells and tissues. Nat Methods 16: 533–544. 10.1038/s41592-019-0404-0 31110282 PMC6544483

[GAD348234MANC10] Lafontaine DLJ, Riback JA, Bascetin R, Brangwynne CP. 2020. The nucleolus as a multiphase liquid condensate. Nat Rev Mol Cell Biol 22: 165–182. 10.1038/s41580-020-0272-6 32873929

[GAD348234MANC11] Liu P, Siciliano J, Seong D, Craig J, Zhao Y, de Jong PJ, Siciliano MJ. 1993. Dual Alu polymerase chain reaction primers and conditions for isolation of human chromosome painting probes from hybrid cells. Cancer Genet Cytogenet 65: 93–99. 10.1016/0165-4608(93)90213-6 8453610

[GAD348234MANC12] Mangan H, Gailín MO, McStay B. 2017. Integrating the genomic architecture of human nucleolar organizer regions with the biophysical properties of nucleoli. FEBS J 284: 3977–3985. 10.1111/febs.14108 28500793

[GAD348234MANC13] McStay B. 2016. Nucleolar organizer regions: genomic ‘dark matter’ requiring illumination. Genes Dev 30: 1598–1610. 10.1101/gad.283838.116 27474438 PMC4973289

[GAD348234MANC14] Meguro-Horike M, Horike S. 2015. MMCT-mediated chromosome engineering technique applicable to functional analysis of lncRNA and nuclear dynamics. Methods Mol Biol 1262: 277–289. 10.1007/978-1-4939-2253-6_17 25555588

[GAD348234MANC15] Misteli T. 2020. The self-organizing genome: principles of genome architecture and function. Cell 183: 28–45. 10.1016/j.cell.2020.09.014 32976797 PMC7541718

[GAD348234MANC16] Peltonen K, Colis L, Liu H, Trivedi R, Moubarek MS, Moore HM, Bai B, Rudek MA, Bieberich CJ, Laiho M. 2014. A targeting modality for destruction of RNA polymerase I that possesses anticancer activity. Cancer Cell 25: 77–90. 10.1016/j.ccr.2013.12.009 24434211 PMC3930145

[GAD348234MANC17] Potapova TA, Unruh JR, Yu Z, Rancati G, Li H, Stampfer MR, Gerton JL. 2019. Superresolution microscopy reveals linkages between ribosomal DNA on heterologous chromosomes. J Cell Biol 218: 2492–2513. 10.1083/jcb.201810166 31270138 PMC6683752

[GAD348234MANC18] Ramlee MK, Yan T, Cheung AM, Chuah CT, Li S. 2015. High-throughput genotyping of CRISPR/Cas9-mediated mutants using fluorescent PCR-capillary gel electrophoresis. Sci Rep 5: 15587. 10.1038/srep15587 26498861 PMC4620477

[GAD348234MANC19] Raška I, Shaw PJ, Cmarko D. 2006. Structure and function of the nucleolus in the spotlight. Curr Opin Cell Biol 18: 325–334. 10.1016/j.ceb.2006.04.008 16687244

[GAD348234MANC20] Sullivan GJ, Bridger JM, Cuthbert AP, Newbold RF, Bickmore WA, McStay B. 2001. Human acrocentric chromosomes with transcriptionally silent nucleolar organizer regions associate with nucleoli. Embo J 20: 2867–2877. 10.1093/emboj/20.11.2867 11387219 PMC125486

[GAD348234MANC21] Sun X, Kaufman PD. 2018. Ki-67: more than a proliferation marker. Chromosoma 127: 175–186. 10.1007/s00412-018-0659-8 29322240 PMC5945335

[GAD348234MANC22] van Sluis M, McStay B. 2015. A localized nucleolar DNA damage response facilitates recruitment of the homology-directed repair machinery independent of cell cycle stage. Genes Dev 29: 1151–1163. 10.1101/gad.260703.115 26019174 PMC4470283

[GAD348234MANC23] van Sluis M, McStay B. 2019. Nucleolar DNA double-strand break responses underpinning rDNA genomic stability. Trends Genet 35: 743–753. 10.1016/j.tig.2019.07.001 31353047

[GAD348234MANC24] van Sluis M, Gailín MO, McCarter JGW, Mangan H, Grob A, McStay B. 2019. Human NORs, comprising rDNA arrays and functionally conserved distal elements, are located within dynamic chromosomal regions. Genes Dev 33: 1688–1701. 10.1101/gad.331892.119 31727772 PMC6942050

[GAD348234MANC25] van Sluis M, van Vuuren C, Mangan H, McStay B. 2020. NORs on human acrocentric chromosome p-arms are active by default and can associate with nucleoli independently of rDNA. Proc Natl Acad Sci 117: 10368–10377. 10.1073/pnas.2001812117 32332163 PMC7229746

[GAD348234MANC26] Weil TT, Parton RM, Davis I. 2010. Making the message clear: visualizing mRNA localization. Trends Cell Biol 20: 380–390. 10.1016/j.tcb.2010.03.006 20444605 PMC2902723

[GAD348234MANC27] Yang L, Guell M, Byrne S, Yang JL, De Los Angeles A, Mali P, Aach J, Kim-Kiselak C, Briggs AW, Rios X, 2013. Optimization of scarless human stem cell genome editing. Nucleic Acids Res 41: 9049–9061. 10.1093/nar/gkt555 23907390 PMC3799423

[GAD348234MANC28] Yao RW, Xu G, Wang Y, Shan L, Luan PF, Wang Y, Wu M, Yang LZ, Xing YH, Yang L, 2019. Nascent pre-rRNA sorting via phase separation drives the assembly of dense fibrillar components in the human nucleolus. Mol Cell 76: 767–783.e11. 10.1016/j.molcel.2019.08.014 31540874

