# Supplementary material for: Human nucleoli comprise multiple constrained territories, tethered to individual chromosomes
Source: Genes Dev. 2021 Apr 1;35(7-8):483–8. doi: 10.1101/gad.348234.121 (PMC8015717; doi:10.1101/gad.348234.121)
Supplement: Supplemental Material [file supp_35_7-8_483__index.html]

Human nucleoli comprise multiple constrained territories, tethered to individual chromosomes — Supplemental Material 

# Human nucleoli comprise multiple constrained territories, tethered to individual chromosomes

## Supplemental Material

- Supplemental\_Data.pdf
