# Supplementary material for: Human nucleoli comprise multiple constrained territories, tethered to individual chromosomes
Source: Genes Dev. 2021 Apr 1;35(7-8):483–8. doi: 10.1101/gad.348234.121 (PMC8015717; doi:10.1101/gad.348234.121)
Supplement: Supplemental Material [file supp_gad.348234.121_Supplemental_Data.pdf]

Human nucleoli comprise multiple constrained territories, tethered to individual chromosomes

Hazel Mangan and Brian McStay

List of Supplemental Material

Supplemental Materials and Methods
Supplemental References
Supplemental Figures S1-S9
Supplemental Table S1-S2

Supplemental Materials and Methods

Plasmids

To construct pDEST N-mAG-MS2 (neo) the MCP orf was cloned in to Gateway™ entry vector pENTR4 (Invitrogen) then transferred into pDEST N-mAG (neo), a custom made vector, using Gateway LR Clonase™(Invitrogen). For pDEST Nop52-mCherry C-term (neo), a Nop52 orf in pENTR4 Nop52 was transferred into custom made vector pDEST mCherry C-term (neo) using Gateway LR Clonase™. To construct pDEST N-mAG-MS2 (neo) the MS2 bacteriophage coat protein (MCP) orf was synthesised (GenScript) and cloned in to Gateway™ entry vector pENTR4 then transferred into pDEST N-mAG (neo) using Gateway LR Clonase™.

The plasmid pT7-Puro was constructed by sub-cloning a HindIII-NheI fragment from the vector pBABE-puro into HindIII-XbaI digested pBluescriptII SK(+). For Puro mRNA synthesis, NotI linearised pT7-puro was transcribed, capped and poly A-tailed using mMESSAGE mMACHINE™ T7 Transcription and Poly(A) Tailing kits (Invitrogen).

gRNA synthesis

Most gRNAs were produced by T7 RNA polymerase transcription of templates prepared by PCR. Transcription templates were prepared in a 2-step PCR process. Firstly, a universal scaffold was prepared using ScaExF and T7gRNArev primers with an stem extension (Ex) and A–U flip (F) and modified gRNA empty vector as template (van Sluis and McStay 2015). T7 promoters and specific gRNA sequences were added by further PCR using the scaffold as template. gRNA templates were *in vitro* transcribed using the

MEGAscript™ T7 Transcription Kit. PCR primer sequences are presented in Supplemental Table S1.

Generation of hTERT-RPE1 Hyg^{-ve} cells

4μg of gRNA targeting immediately downstream of the start codon of the HygR orf in the hTERT expression plasmid pGRN145 was pre-complexed with 10μg Cas9 for 10mins at room temperature. RNP complexes were electroporated into 10^6 hTERT-RPE1 cells using a Nucleofector 2b, the programme T-020 and Nucleofector kit V (Lonza). The clone selected has a 23nt deletion verified (Supplemental Fig. S1). To aid selection during MMCT, cells were subsequently transfected with BSR plasmid pJRC41 and selected in 20μg/ml blastcidin (Melford).

Ribosome and Polysome purification

Purification of cytoplasmic ribosomes was performed from 5 x 15cm dishes of hTERT-RPE1 and derivative cells as described previously (Belin et al. 2010). Ribosomal RNA was purified using a NucleoSpin RNA II kit (Machery-Nagel). RNA was eluted in nuclease-free water and stored at -80°C.

For polysome purification, 5 x 15cm plates of hTERT-RPE1 and derivative cells were grown to 80% confluency. Cell were treated with 100 μg/mL cycloheximide (Sigma) for 15 minutes prior to harvesting. All washes were preformed ice-cold. Cells were washed with PBS containing 100 μg/mL cycloheximide and 1 mM ribonucleoside vanadyl complexes (Sigma). Cells were harvested by scraping into the same solution and pelleted by centrifugation at 500g for 5 min at 4°C. Cells were lysed in (0.5% Igepal, 100 μg/mL cycloheximide, 5 mM DTT, 300 mM KCl, 5 mM MgCl2, 10 mM HEPES,

10 mM ribonucleoside vanadyl complexes) and passaged 20 times through a 25G syringe needle. Nuclei were removed by centrifugation at 12,000g for 10 min at 4°C. The cytoplasmic fraction was carefully loaded onto a 15-45% sucrose gradient in 300 mM KCl, 5 mM MgCl2, 10 mM HEPES, 10 mM ribonucleoside vanadyl complexes prepared using a Gradient Master (BioComp). Following ultracentrifugation at 43,500 rpm for 50 min at 4°C in a Beckman SW50/55 rotor, fractions were collected by piercing the bottom of the gradient with a 19G syringe needle. 15µl of each fraction was loaded directly on an agarose gel to determine the profile. Polysome containing fractions were pooled and RNA was purified using NucleoSpin RNA II kit as above.

RNA Analysis by Northern Blotting and RT-PCR

RNA was separated by agarose-formaldehyde gel electrophoresis in 3-(N-morpholino)propanesulfonic acid (MOPS) buffer, equilibrated and blotted onto Nylon N+ membrane (Amersham) by capillary transfer in 10X UltraPure SSC. RNA was crosslinked by 70,000 µJ/cm2 UV irradiation. A pre-hybridisation wash was performed in hybridization buffer (0.5M phosphate buffer pH 7.2, 1% BSA, 7% SDS, 1mM EDTA) for 30 mins at 60°C. 250ng of a digoxigenin (DIG) labelled oligonucleotide probe, DIG-28Sr (Supplemental Table S1), was denatured by boiling for 5 mins before adding to the fresh hybridization buffer. Post-hybridisation washes were 5 mins in 2X SSC/0.1% SDS, 15 mins in 1X SSC/0.1% SDS and 20 mins in 0.1X SSC/0.1% SDS all at 65°C. DIG was detected using anti-Digoxigenin-POD, Fab fragments and a CDP-Star detection kit (Roche). Signal was detected by chemiluminescence using

WesternBright ECL (Advansta) and a C-DiGit imager (Licor). RT-PCR was performed using an RT-PCR Luna kit (NEB).

Supplemental References

Belin S, Hacot S, Daudignon L, Therizols G, Pourpe S, Mertani HC, Rosa-Calatrava M, Diaz JJ. 2010. Purification of ribosomes from human cell lines. *Curr Protoc Cell Biol* **Chapter 3**: Unit 3 40.

van Sluis M, McStay B. 2015. A localized nucleolar DNA damage response facilitates recruitment of the homology-directed repair machinery independent of cell cycle stage. *Genes Dev* **29**: 1151-1163.

hTERT-RPE1

TCCGGGAGAT**ATG**AAAAAGCCTGAACTCACCGCGACGTCTGTCGAGAAGTTTCTGATCGA **HygR (WT)**
AGGCCCTCTATACTTTTTC**GGA**CTTGAGTGGCGCTGCAGACAGCTCTTCAAAGACTAGCT
PAM gRNA

hTERT-RPE1 Hyg^{-ve}

TCCGGGAGAT**ATG**AAACAAGCTGAA----- 23nt deletion --------GTTTCTGATCGA **HygR (KO)**
AGGCCCTCTATACTTTTTC**GGA**CTT----- --------CAAAGACTAGCT

Supplemental Fig. S1. Generation of hTERT-RPE1 Hyg^{-ve} MMCT recipient cells. The sequence surrounding the translation initiation codon (green) of the hygromycin resistance (HygR) orf present in the hTERT plasmid used to immortalise RPE1 cells. The sequence of the gRNA is underlined and the PAM site is in red. The sequence from the HygR knockout (KO) cell line, with its 23nt deletion is shown below.

A

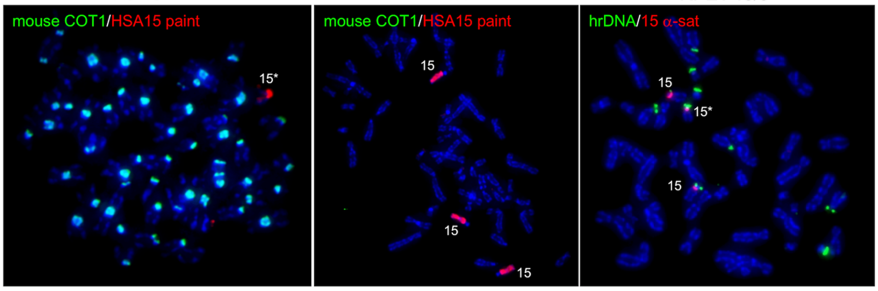

B

RPE1 15/3

Human chromosome	Marker	Allele 1	Allele 2	Allele 3
5	CSF1PO	12	14	
13	D13S317	11	12	
16	D16S539	11	11	
18	D18S51	14	16	
21	D21S11	31	32.2	
3	D3S1358	15	16	
5	D5S818	11	11	
7	D7S820	10	11	
8	D8S1179	10	10	
4	FGA	20	23	
21	Penta D	9	11	
15	Penta E	12	14	10
11	TH01	9	9	
2	TPOX	8	8	
12	vWA	17	18	
X/Y	AMEL	X	X	

Supplemental Fig. S2 Validation of MMCT protocol. (A) Metaphase-FISH confirms transfer of HSA15 from A9-15 to hTERT-RPE1-Hyg-ve cells generating RPE1 15/3. Human rDNA was visualised using a human specific IGS probe, mouse chromosomes by mouse Cot-1 DNA and HSA15 by either chromosome paint or a HSA15 specific α-satellite centromere probe. The transferred HSA15, identified by proportional rDNA content, is indicated by an asterisk. (B) STR profiling of RPE1 15/3 using Promega PowerPlex STR 16HS and performed by Source BioSciences UK, note the third allele for HSA15 STR marker Penta E.

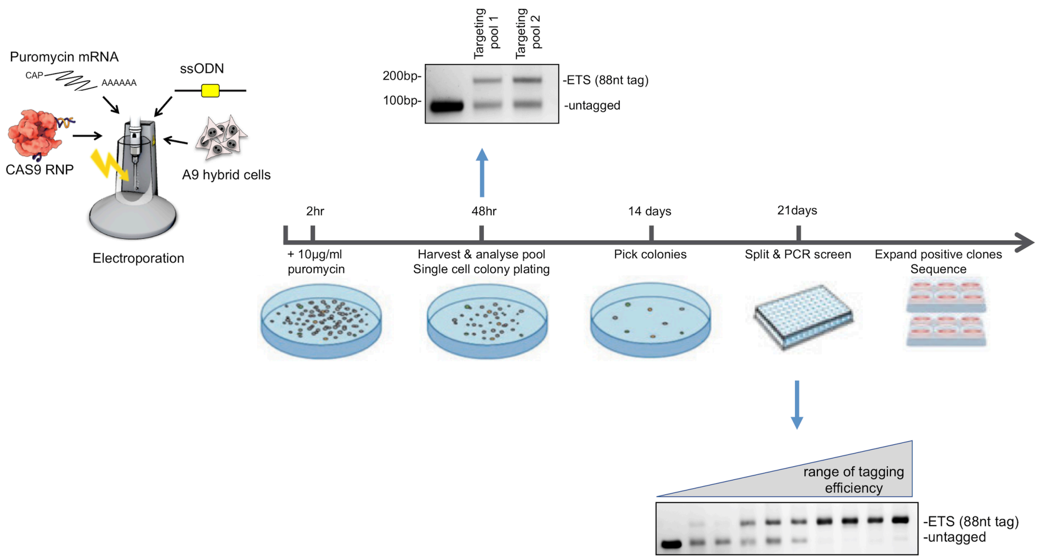

Supplemental Fig. S3. NOR Editing workflow. The workflow is presented diagrammatically. Results from PCRs performed on transfected pools and selected clones are also shown. In the targeting experiment shown, an 88nt tag was introduced into the 5'ETS of human rDNA repeats in the HSA15 present in A9-15. Selected clones range from low to >90% targeting efficiency. See Methods for details.

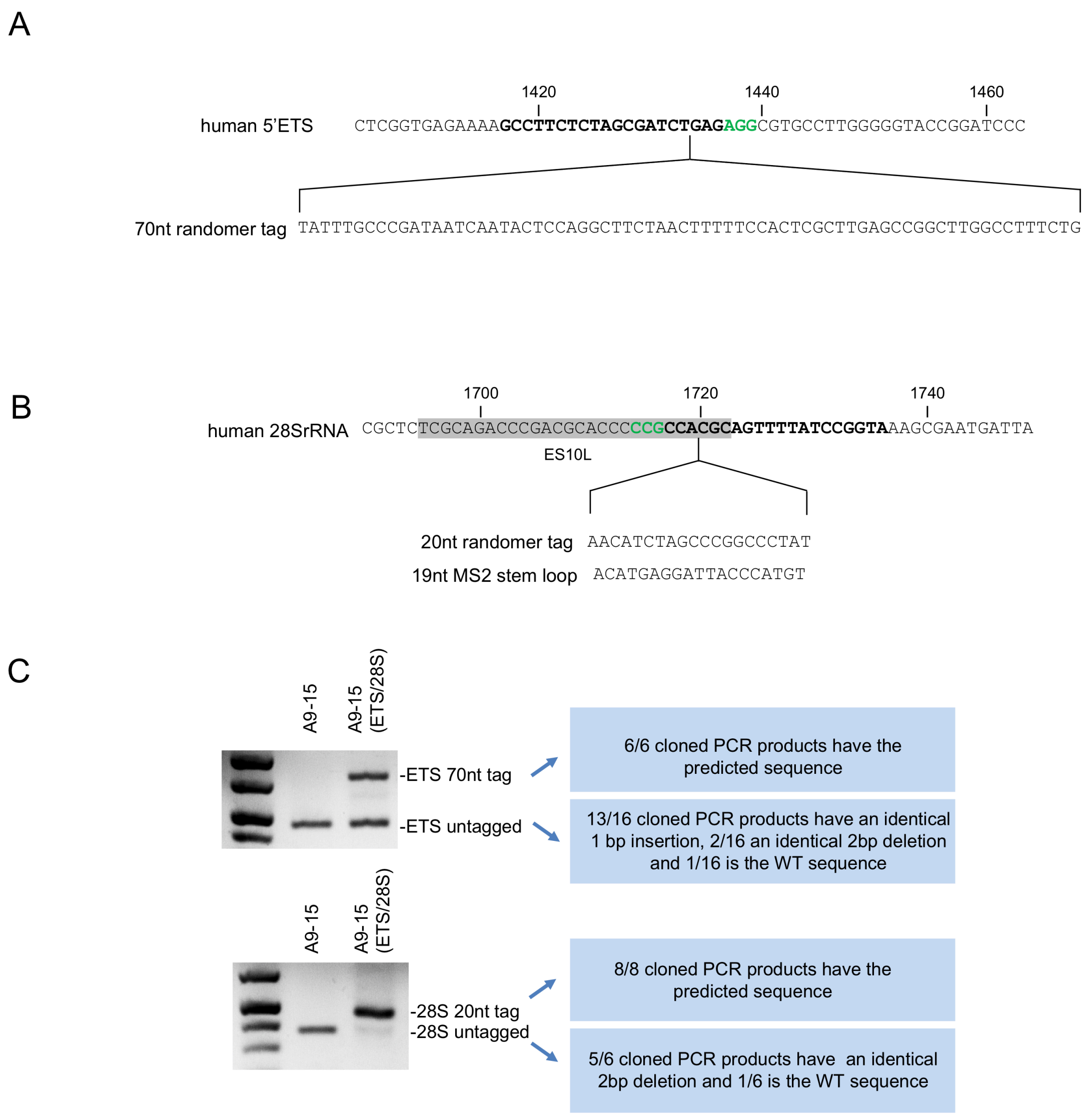

Supplemental Fig. S4. Sequence tagging of pre-rRNA coding sequences. (A) DNA sequence surrounding the 5'ETS tagging site. Coordinates are shown above, gRNA sequence and PAM site are in bold and green respectively. The sequence of the 70nt randomer tag is shown below. (B) DNA sequence surrounding the 28SrRNA tagging site. Expansion segment 10L (ES10L) of human 28SrRNA is shaded grey. The sequences of 20nt randomer and 19nt MS2 stem loop tags are shown below. (C) DNA sequencing of cloned PCR products spanning 5'ETS and 28S tagging sites in A9-15 (ETS/28S). The sequences of tagged repeats are uniformly identical to predicted outcomes. The majority of non-tagged repeats show evidence of DNA DSB and repair by the NHEJ pathway, i.e. the presence of indels.

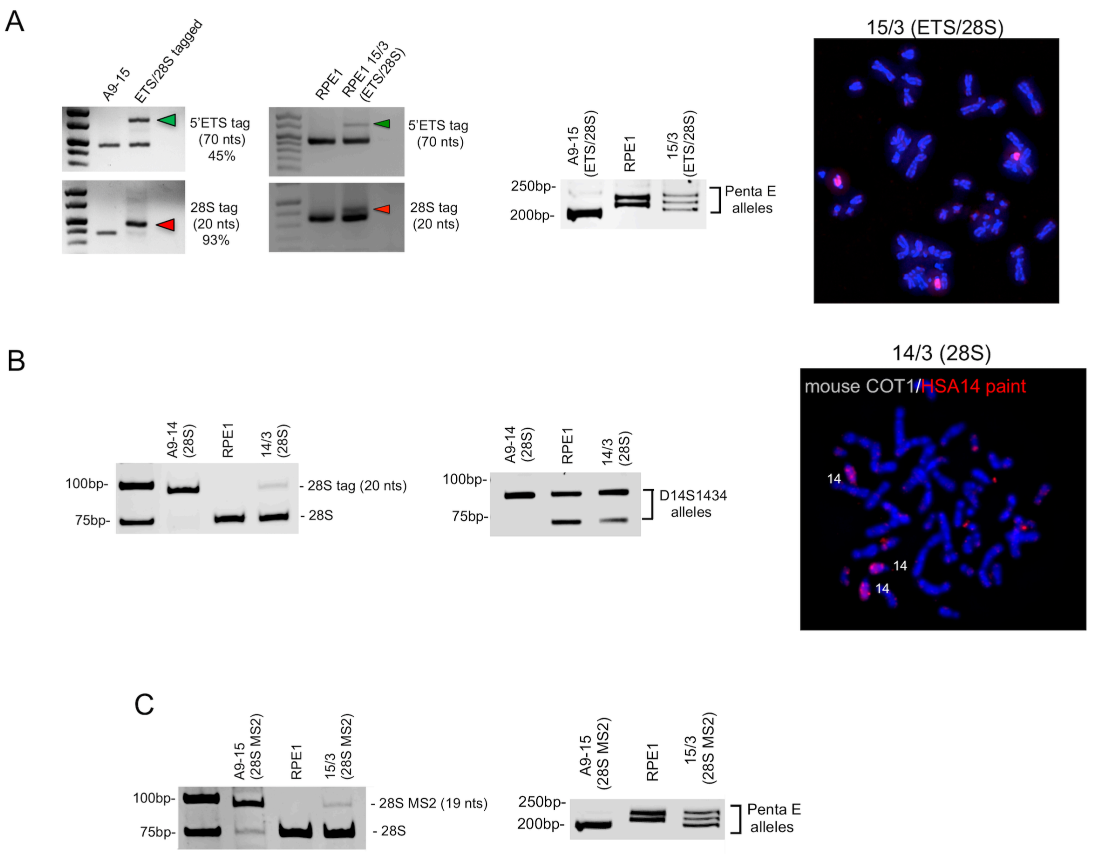

Supplemental Fig. S5. Evidence supporting sequence tagging in hybrid cells and MMCT to hTERT-RPE1-Hyg^{-ve} cells. (A) PCR reveals the efficiency tagging at 5'ETS and 28S cells in the A9-15 hybrid and the proportion of tagged repeats in RPE1 15/3 (ETS/28S) after MMCT. PCR across the HSA15 Penta E STR and metaphase FISH confirm the transfer of the tagged HSA15 in RPE1 15/3 (ETS28S). (B) PCR reveals >90% efficiency of 28S tagging in the A9-14 hybrid and the proportion of tagged repeats in RPE1 14/3 (28S) after MMCT. PCR across the HSA14 D14S1434 STR and metaphase FISH confirm the transfer of the tagged HSA14 in RPE1 14/3 (28S). Note the doubled intensity of the upper D14S1434 allele in RPE1 14/3 (28S). (C) PCR reveals efficiency of 28SMS2 tagging in the A9-154 hybrid and the proportion of tagged repeats in RPE1 15/3 (28SMS2) after MMCT. PCR across the HSA15 Penta E STR confirms the transfer of the tagged HSA15 in RPE1 15/3 (28SMS2)

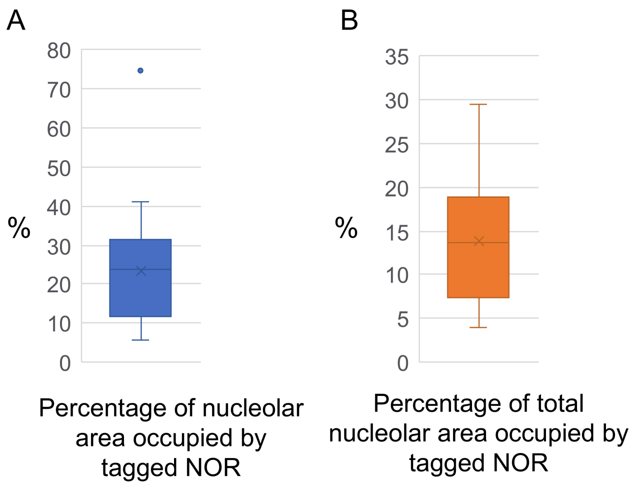

Fig. S6. The proportion of nucleolar area occupied by tagged NORs
(A) To determine the percentage of nucleolar area occupied by tagged NORs, data from the experiment presented in Fig. 2C were analysed. The area of the tagged NOR, visualised by 28S tag hybridisation signal, was determined as a percentage of the area of the same nucleolus, visualised by 18SE probe hybridisation signal. 25 cells were analysed and the data presented as a box and whisker plot. The outlier is small a nucleolus, presumed to comprise only the tagged NOR **(B)** As above except that the area of the tagged NOR is expressed as a percentage of the summed area of all the nucleoli in that cell.

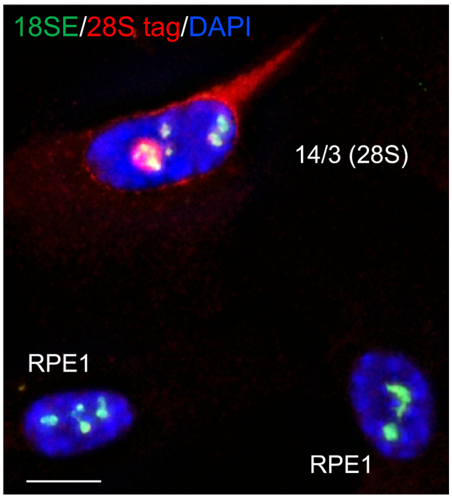

Supplemental Fig. S7. Validation of 28S tag probe. RNA-FISH was performed on co-cultured 14/3 (28S) and hTERT-RPE1 cells. Probes were the 28S-tag oligo probe (red) and the 18SE probe (green). Note the absence of tag hybridisation in RPE1 cells. Scale bar 10 μm.

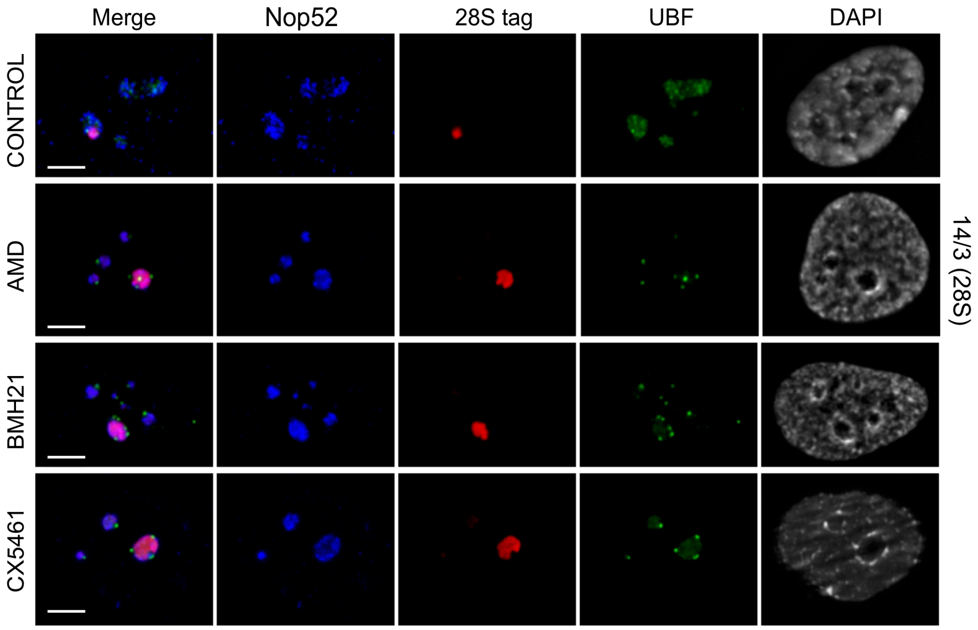

Supplemental Fig. S8. Loss of NOR territories in 14/3 (28S) cells during nucleolar stress. ImmunoRNA-FISH was performed on 14/3 (28S) cells either untreated (control) or treated with AMD, BMH21 or CX5461. Nucleoli were visualised using antibodies against Nop52 and UBF (GC and FC marker proteins respectively). Scale bars 5 μm.

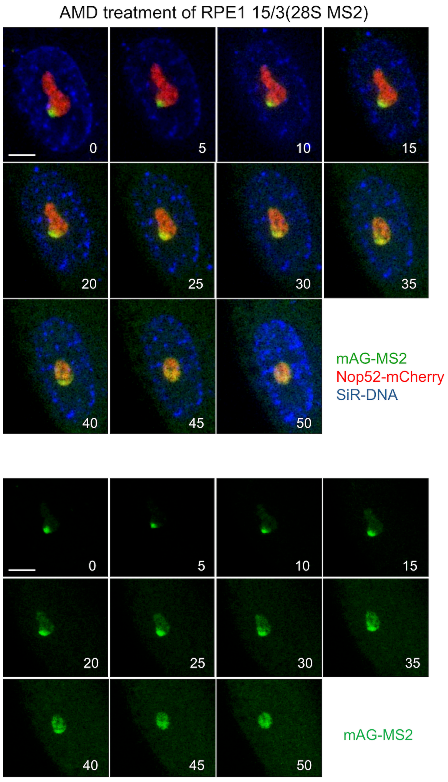

Supplemental Fig. S9. Imaging blending of NOR territories during nucleolar stress in live cells. RPE1 15/3 (28SMS2) cells were first transfected with plasmids expressing Nop52-mCherry (red) and mAG-MS2 fusion proteins. Live cells were then treated with AMD and imaged at 5 min intervals thereafter. DNA was visualised in live cells using SiR-DNA (pseudo-coloured in blue). The upper panels show merged images at 5 minute intervals, while those below show only mAG-MS2. Note the progressive spreading of mAG-MS2 throughout the nucleolar volume over the 50 minute imaging period. Scale bars 5 μm.

**Oligonucleotides (supplied by Integrated DNA Technologies)**		
**PCR primers used in gRNA synthesis**		
T7g5’ETS	GGATCCTAATACGACTCACTATA*GCCTTCTCTAGCGATCTGAG*GTTTAA<br>GAGCTATGCTG	Synthesis of 5’ETS gRNA
T7g28S	GGATCCTAATACGACTCACTATA*GACCGGATAAAACTGCGTGG*GTTTAA<br>GAGCTATGCTG	Synthesis of 28S gRNA
T7gHYG	GGATCCTAATACGACTCACTATA*GCAGACGTCGCGGTGAGTTC*GTTTA<br>AGAGCTATGCTG	Synthesis of Hyg gRNA
ScafexF	GTTTAAGAGCTATGCTGGAAAC	
t7gRNArev	AAAAGCACCGACTCGGTGCC	
**Ultramer® DNA Oligo**		
5’ETSr TAG	GCGAGCGGGTCGCTACGGCAGCGCTACCATAACGGAGGCAGAGACA<br>GAGGCGGCGGCCCGGGGGATCCGGTACCCCCAAGGCACGCCTCTCCA<br>GAAAGGCCAAGCCGGCTCAAGCGAGTGGAAAAAGTTAGAAGCCTGG<br>AGTATTGATTATCGGGCAAATAAGATCGCTAGAGAAGGCTTTTCTCAC<br>CGAGGGTGGG	SsODN for introducing 70nt 5’ETS tag
28Sr TAG	TCGCAGACCCGACGCACCCCCGCCAAACATCTAGCCCGGCCCTATCG<br>CAGTTTTATCCGGTAAAGCGAAT	SsODN for 20nt 28S tag
28S MS2	TCGCAGACCCGACGCACCCCCGCCAACATGAGGATCACCCATGTCGC<br>AGTTTTATCCGGTAAAGCGAAT	SsODN for 19nt 28S MS2 tag
**Oligonucleotide probes**		
5'ETSr TAG	/5Alex488N<br>CTCTCCAGAAAGGCCAAGCCGGCTCAAGCGAGTGGAAAAAG<br>/5Alex488N TTAGAAGCCTGGAGTATTGATTATCGGGCAAATAAGATCG	Two RNA-FISH probes for 5’ETS tag
g28Sr TAG	/5’Alex594N CTGCGATAGGGCCGGGCTAGATGTTTG<br>/5’DiGN CTGCGATAGGGCCGGGCTAGATGTTTG	RNA-FISH and northern probes for 28S tag
H18SE	/5’Alex594N CGGGCCTCGCCCTCCGGGCTCCGTTAATG<br>/5Alex488N CGGGCCTCGCCCTCCGGGCTCCGTTAATG	RNA-FISH probes for endogenous 18SE pre-rRNA
**Standard PCR primers**		
PCR across 5’ETS tag site	F AGGAGGAGCGGCTGGC<br>R GGGAAGCGGAGGAGGGT<br>F ACCCTCGGTGAGAAAAGCCT<br>R GCAGCGCTACCATAACGGA	Set1<br><br>Set2
PCR across 28S tag site	F CTCTCGCAGACCCGACG<br>R CGGCCCCAAGACCTCTAATC<br>Alternate reverse primer<br>R CACTAGGCACTCGCATTCCA	
PCR across HYG Cas9 cleavage site	F TCCTCGGCCCAAAGCATCAG<br>R CAGCGTCTTGTCATTGGCGA	
PentaE (15 STR)	F GCTACTCTGGAGGCTGAAACA<br>R TGGGTTATTAATTGAGAAAACTCCTTACAATTT	Used in STR genotyping for HSA15
D14S1434 (14 STR)	F ACTCTACGACTGTCTGTCTG<br>R CAATAGGAGGTGGATGGATG	Used in STR genotyping for HSA14
**Primers for chromosome paints**		
Inter-Alu primers	F GGATTACAGGYRTGAG*C*C*A<br>R RCCAYTGCACTCCAGC*C*T*G	*phosphorothioate
**Oligos for Generating SABER FISH probe**		
G2R26.1	CGATCTTATTTGCCCGATAATCAATACTCCAGGCTTCTAATTTATAAA<br>CCTA	
G2R26.2	TTTTCCACTCGCTTGAGCCGGCTTGGCCTTTCTGGAGAGGTTTATAAA<br>CCTA	

Supplemental Table. S1 Oligonucleotides used in this study

**Antibodies**		
NOP52 (rabbit)	Novus	Cat#NBP1-85338
NOP52 (sheep)	McStay lab	N/A
UBF (sheep)	McStay lab	N/A
FIBRILLARIN (mouse) Clone 72B9	Cytoskeleton, Inc.	Cat#AFB01
NUCLEOLIN (sheep)	McStay lab	N/A
Anti-mouse Alexa Flour® 594	Jackson Immuno Research (JIR)	Cat#715-585-150
Anti-sheep Alexa Flour® 488	JIR	Cat#713-545-147
Anti-sheep Alexa Flour® TRITC	JIR	Cat#713-025-147
Anti-sheep Alexa Flour® 647	JIR	Cat#713-605-147
Anti-sheep DyLight ™ 405	JIR	Cat#713-475-147
Anti-rabbit TRITC	JIR	Cat#711-025-152
Anti-rabbit Alexa Flour® 594	JIR	Cat#711-585-152

Supplemental Table. S2 Antibodies used in this study
